# Genetic dissection of medial habenula–interpeduncular nucleus pathway function in mice

**DOI:** 10.3389/fnbeh.2013.00017

**Published:** 2013-03-12

**Authors:** Yuki Kobayashi, Yoshitake Sano, Elisabetta Vannoni, Hiromichi Goto, Hitomi Suzuki, Atsuko Oba, Hiroaki Kawasaki, Shigenobu Kanba, Hans-Peter Lipp, Niall P. Murphy, David P. Wolfer, Shigeyoshi Itohara

**Affiliations:** ^1^Laboratory for Behavioral Genetics, RIKEN Brain Science InstituteSaitama, Japan; ^2^Institute of Anatomy, University of ZürichZürich, Switzerland; ^3^Institute of Human Movement Sciences and Sport, ETH ZurichZürich, Switzerland; ^4^Department of Neuropsychiatry, Graduate School of Medical Sciences, Kyushu UniversityFukuoka, Japan; ^5^Departments of Psychiatry and Biobehavioral Sciences, Semel Institute for Neuroscience and Human Behavior, University of CaliforniaLos Angeles, CA, USA

**Keywords:** colinergic circuit, diphthelia toxin, genetic cell abration, decision making, maladaptation, impulsivity and self-control, schizhophrenia, ADHD

## Abstract

The habenular complex linking forebrain and midbrain structures is subdivided into the medial (mHb) and the lateral nuclei (lHb). The mHb is characterized by the expression of specific nicotinic acetylcholine receptor isoforms and the release of acetylcholine to the interpeduncular nucleus (IPN), the sole output region of the mHb. The specific function of this circuit, however, is poorly understood. Here we generated transgenic mice in which mHb cells were selectively ablated postnatally. These lesions led to large reductions in acetylcholine levels within the IPN. The mutant mice exhibited abnormalities in a wide range of behavioral domains. They tended to be hyperactive during the early night period and were maladapted when repeatedly exposed to new environments. Mutant mice also showed a high rate of premature responses in the 5-choice serial reaction time task (5-CSRTT), indicating impulsive and compulsive behavior. Additionally, mice also exhibited delay and effort aversion in a decision-making test, deficits in spatial memory, a subtle increase in anxiety levels, and attenuated sensorimotor gating. IntelliCage studies under social housing conditions confirmed hyperactivity, environmental maladaptation, and impulsive/compulsive behavior, delay discounting, deficits in long-term spatial memory, and reduced flexibility in complex learning paradigms. In 5-CSRTT and adaptation tasks, systemic administration of nicotine slowed down nose-poke reaction and enhanced adaptation in control but not mutant mice. These findings demonstrate that the mHb–IPN pathway plays a crucial role in inhibitory control and cognition-dependent executive functions.

## Introduction

The habenular complex of the epithalamus anatomically and functionally links forebrain and midbrain structures (Klemm, 2004; Lecourtier and Kelly, 2007). The medial nucleus (mHb) receives septal inputs and sends efferents solely to the interpeduncular nucleus (IPN), while the lateral nucleus (lHb) receives limbic and pallidal inputs, and sends efferents to monoaminergic systems, such as the ventral tegmental area (VTA) and raphe nuclei (Klemm, 2004; Lecourtier and Kelly, 2007). The habenula is heterogeneous, even within the lHb and mHb subdivisions (Andres et al., 1999; Kim and Chang, 2005). Cells in the ventral mHb release acetylcholine to the IPN (Grady et al., 2009), while cells in the dorsal mHb express substance P (SP) (Mroz et al., 1976; Cuello et al., 1978). Some axonal branches originating from forebrain areas pass through the habenular complex to the midbrain/hindbrain areas, further increasing the complexity of the habenula (Contestabile and Flumerfelt, 1981; Albanese et al., 1985).

Imaging and histopathologic studies in humans suggest habenular complex dysfunction as a pathologic mechanism in some mental disorders, such as schizophrenia and depression (Sandyk, 1992; Caputo et al., 1998; Ranft et al., 2010). Classic lesion studies in rodents suggest crucial roles for the habenular complex in various behavioral domains, such as emotion, learning and memory, and impulsivity, and support the involvement of habenular dysfunction in mental disorders (Thornton et al., 1990; Murphy et al., 1996; Amat et al., 2001; Klemm, 2004; Lecourtier et al., 2004, 2005; Lecourtier and Kelly, 2005, 2007; Heldt and Ressler, 2006). The small and complex habenular structure makes it difficult to identify the roles of the subnuclei. Although recent physiologic studies in monkeys provided the first insight into how the lHb functions in reward processing and punishment prediction (Matsumoto and Hikosaka, 2007, 2009; Hikosaka et al., 2008), the function of the mHb has been largely overlooked. A recent study in zebrafish demonstrated that the lateral region of the dorsal subnucleus of the habenula, a potential counterpart of the mHb in mammals, is involved in experience-dependent fear responses (Agetsuma et al., 2010). Medial nuclei cells are molecularly characterized by the high expression of unique nicotinic acetylcholine receptors containing α3,α5, and β4 subunits (Xu et al., 2006; Grady et al., 2009), and their involvement is implicated in nicotine's reinforcing effects (McCallum et al., 2012) and nicotine withdrawal symptoms in mice (Salas et al., 2009). Genetic manipulation of the α5 subtype in mHb cells revealed a role for the mHb–IPN pathway in limiting nicotine intake (Fowler et al., 2011; Frahm et al., 2011).

Genetic methods provide an invaluable advantage for analyzing the differential roles of complex circuits. Utilizing these techniques in mice, we analyzed the mHb–IPN pathway and demonstrated its crucial role in various behavioral domains, particularly inhibitory control and cognition-dependent executive functions.

## Materials and methods

All experimental protocols were approved by the Animal Care and Use Committees of the RIKEN Brain Science Institute and Veterinary Office of the Canton of Zurich.

### Animals

#### Generation of mHB:DTA transgenic mice

A bacterial artificial chromosome clone (MSMg01-81G4) containing *Gpr151 (PGR7*, *GalRL*, and *GPCR2037)* was obtained from the RIKEN BioResource Center. The nuclear localization sequence (NLS)-Cre-pA cassette was inserted downstream of the *Gpr151* promoter using the Red/ET recombination technique (Gene Bridges, Dresden, Germany). This vector was injected into C57BL/6-fertilized eggs and the resulting *Gpr151*-Cre mice were maintained on a C57BL/6 background. Transgenic mice were genotyped by polymerase chain reaction (PCR), using the following primers: CW-Cre2, 5′-ACC TGA TGG ACA TGT TCA GGG ATC G-3′ and CW-Cre3, 5′-TCC GGT TAT TCA ACT TGC ACC ATG C-3′, producing a 108-base pair (bp) fragment from the Cre allele.

*Eno2*-Diphtheria toxin A (DTA) mice (Kobayakawa et al., 2007), which contain the IRES-loxP-STOP(pgk-neo-polyA)-loxP-DTA cassette at the ApaI site in the 3′-untranslated region of the mouse *Eno2* gene, were maintained as a C57BL/6 congenic strain. *Eno2* promoter activity and Cre-mediated recombination allow for expression of the DTA fragment in selected mature neurons. DTA has ADP ribosyltransferase activity that inhibits elongation factor 2 and thus induces apoptotic cell death (Kobayakawa et al., 2007). Mouse genotypes were determined by PCR using the following primers: NDp1, 5′-AAT TCT TAA TTA AGG CGC GCC GG-3′; NDp2, 5′-GTC AGA ATT GAG GAA GAG CTG GGG-3′; and NDp3, 5′-CAC TGA GGA TTC TTC TGT GG-3′. Fragments of 378 bp and 294 bp were amplified from wild-type and knock-in alleles, respectively.

The double transgenic *(Eno2-DTA:Gpr151-Cre)* mice were designated mHb:DTA mice. Both male and female mice were used for histologic and neurochemical analyses, and no sex differences were detected. Wild-type littermates were used as controls for tyrosine hydroxylase (TH) immunohistochemistry and monoamine and acetylcholine measurements. DTA single transgenic mice were used as genetic controls for histologic experiments and conventional behavioral tests. Male mice 4–8 months of age were analyzed using conventional behavioral experiments. Female mice 4–5 months of age at the onset of testing were used for the IntelliCage experiments. In pharmacologic and c-Fos mapping experiments, we used wild-type males as genetic controls.

#### Rosa-NLSLacZ (RNZ) and rosa-GAPLacZ (RGZ) mice

According to previously described procedures (Soriano, 1999), a 5-kilobase (kb) fragment of *Rosa26* was used to make the targeting constructs. The splicing acceptor was followed by the loxP-STOP (pgk-Neo-polyA)-loxP and the NLS:LacZ [NLS of the SV40 large T-antigen, followed by the *Escherichia coli* β-galactosidase (β-Gal) gene]-poly(A) or GAP:LacZ [palmitoylation signals of GAP43 (5′-ATG CTG TGC TGT ATG AGA AGA ACC AAA CAG GTT GAA AAG AAT GAT GAG GAC CAA AAG ATC-3′), followed by β-Gal gene]-poly(A) gene cassettes. RNZ and RGN mice were used to facilitate identification of the nuclei and axons, respectively, of Cre-mediated recombinant cells. The targeted ES cell clones were injected into C57BL/6 blastocysts, and the resulting chimeras were crossed with C57BL/6 females to achieve germline transmission. Mouse genotypes were determined by PCR using genomic DNA as a template and the primers R1295: 5′-GCG AAG AGT TTG TCC TCA ACC-3′, R523: 5′-GGA GCG GGA GAA ATG GAT ATG-3′, and R26F2: 5′-AAA GTC GCT CTG AGT TGT TAT-3′. Fragments of 603 bp and 330 bp were amplified from wild-type and knock-in mutant alleles, respectively.

### Histology

#### Measurement of relative neuron numbers

Mice were heavily anesthetized with 2,2,2-tribromoethanol (approximately 500 mg/kg, intraperitoneally, Sigma-Aldrich) and perfused with 4% paraformaldehyde (PFA) in 0.1 M sodium phosphate buffer (PB), pH 7.4, at 4°C for 20 min. The brains were excised, and post-fixed with the same fixative at 4°C overnight. Serial sections of paraffin-embedded brain samples (10 μm) were prepared and stained with cresyl violet. Images were captured using a NanoZoomer Digital Pathology virtual slide scanner (Hamamatsu, Japan). For each mouse, 15 sections at regular intervals were selected throughout the Hb region, corresponding to approximately −1.34 to −2.18 mm from bregma according to the brain atlas of Franklin and Paxinos (1997). The total area of Nissl-stained cells in the mHb and lHb was determined using Image-Pro plus 5.0 J (Media Cybernetics). Signal regions smaller than 50 μm^2^ were excluded from the measurements.

***LacZ staining***. β-Gal activity was detected as described previously (Sano et al., 2009). In brief, mice were deeply anesthetized with 2,2,2-tribromoethanol and transcardially perfused with physiologic saline and then 4% PFA in 0.1 M PB, pH 7.4, at 4°C for 20 min. The brains were excised, post-fixed with the same fixative at 4°C for 2 h, and equilibrated in 30% (w/v) sucrose in PB as a cryoprotectant. The brains were embedded in OCT compound (Sakura Finetech), and frozen sections (20 μm) were prepared. Sections were then washed in phosphate buffered saline (PBS) on ice for 5 min and stained in 1 mg/ml X-gal, 5 mM K3Fe(CN)6, 5 mM K4Fe(CN)6, 20 mM Tris/HCl pH 7.5, and 2 mM MgCl_2_ in PB at 37°C overnight. LacZ-stained sections were washed in PBS and then counter-stained with hematoxylin.

#### In situ hybridization and immunohistochemistry

Brains were excised and post-fixed with 4% PFA at 4°C for 3 days. Coronal sections (80 μm) were prepared using a vibratome. All steps were performed at room temperature (RT) unless indicated otherwise. Sections were incubated with methanol (MeOH) for 2 h, then washed 3 times for 20 min in PBS containing 0.1% Tween-20 (PBST), incubated with 10 μg/ml proteinase K (Invitrogen, Tokyo, Japan) in PBST for 10 min, rinsed in PBST, post-fixed in 4% PFA in 0.1 M PB for 20 min and, finally, washed 3 times for 20 min in PBST. Prior to hybridization, digoxigenin (DIG)-labeled cRNA probes in hybridization buffer (5 × SSC, 50% formamide, 0.1% Tween-20) were denatured at 90°C for 10 min and then quickly cooled on ice for 10 min.

Test cRNA probes were generated using a DIG RNA labeling kit (Roche, Tokyo, Japan). The *Tac1* probe sequence spanned nucleotides 95–995 of GenBank sequence, accession no. NM009311 and the *Chrm2* probe spanned nucleotides 497–1244 of GenBank sequence, accession no. NM203491.

Hybridization was performed at 58°C overnight. Sections were washed in 2 × SSC containing 50% formamide and 0.1% Tween-20 (SSCT) for 60 min, 2 × SSCT for 15 min, 0.2 × SSCT at 58°C for 60 min, and in PBST at RT for 10 min. Sections were incubated overnight with alkaline phosphatase-conjugated anti-DIG antibody (1:2000, Roche) in blocking reagent [1% blocking reagent (Roche), 10 mM maleic acid, 15 mM NaCl, pH 7.5] at 4°C. The sections were washed three times in PBST at RT for 20 min.

For staining with nitroblue tetrazolium chloride/5-bromo-4-chloro-3-indolyl phosphate 4-toluidine salt (NBT/BCIP), the signal was developed in 2% (v/v) NBT/BCIP stock solution (Roche) diluted in staining solution A (0.1 M NaCl, 0.05 M MgCl_2_, 0.1% Tween-20, 0.1 M Tris pH 9.5). For Fast Red staining, the signal was developed in 1 × Fast Red (Roche) diluted in staining solution B (0.1% Tween-20 and 0.1 M Tris pH 8.2).

Sections were further stained with goat polyclonal anti-choline acetyltransferase (ChAT) antibody (1:100, Cat# 144P; Millipore, Billerica, MA) or mouse monoclonal anti-β-Gal (1:1000, Cat# Z378; Promega, Madison, WI). For ChAT staining, the sections were incubated with donkey polyclonal anti-goat IgG, conjugated to Alexa 488 (1:1000, Invitrogen, Tokyo, Japan). For β-Gal staining, the avidin–biotin complex method (Vector Laboratories, Burlingame, CA) was used. The sections were incubated with 0.3% (v/v) hydrogen peroxide in PBS to quench endogenous peroxidase activity and then blocked with 5% (v/v) horse serum in PBS. After overnight incubation at 4°C with primary antibodies, sections were further incubated with horse polyclonal anti-mouse IgG antibody, conjugated to biotin (Vector Laboratories), followed by the avidin–biotin complex. Peroxidase activity was revealed using diaminobenzidine as the chromogen.

To visualize SP, rabbit polyclonal anti-SP (Cat# 20064; ImmunoStar, Hudson, WI) and Alexa 546-conjugated goat polyclonal anti-rabbit IgG (Invitrogen) were used.

#### Quantitative analysis of the IPN area and TH-positive puncta

TH-immunohistochemistry was performed according to a previously described procedure (Battisti et al., 1987), with minor modifications. Free-floating sections (40 μm) were incubated with 25% MeOH in PBS containing 0.1% Tween-20 for 5 min and blocked with 5% normal goat serum in PBS containing 0.3% Tween-20. After incubation overnight at 16°C with mouse monoclonal anti-TH antibodies (1:1000, clone TH-16, Sigma-Aldrich, Tokyo, Japan), the sections were further incubated with Alexa 488-conjugated goat anti-mouse IgG (1:1000, Invitrogen). Stained sections mounted on glass slides were embedded in ProLong Gold Antifade Reagent with DAPI (Invitrogen). Images were captured using a NanoZoomer Digital Pathology virtual slide scanner and confocal fluorescence microscopy (Leica TCS SL, Leica Microsystems, Tokyo, Japan). For each mouse, six sections at 80-μm intervals were selected throughout the IPN region (approximately −3.28 mm from bregma), according to a mouse brain atlas (Franklin and Paxinos, 1997), and both the total area encompassing the IPN and DAPI-stained nuclei in the IPN were measured. The IPN area was identified by TH immunostaining; TH staining is strong in the VTA and SN, thus delineating the IPN. The total area of TH-positive puncta within the IPN was measured using confocal images of six fields, similar to the above analysis. The area of TH-positive puncta was calculated per unit area of a selected field. Signals smaller than 2 μm^2^ and larger than 150 μm^2^ were excluded from measurement. Image-Pro plus 5.0 J (Media Cybernetics) was used for these analyses.

#### Immunohistochemical detection of c-Fos

Rabbit polyclonal anti-cFos (Ab-5) (4-17) (Cat#PC38; Calbiochem, Merck4Biosciences, Tokyo, Japan) was used for cFos detection. Free-floating sections (40 μm) were incubated with 0.3% (v/v) hydrogen peroxide in PBST to quench endogenous peroxidase activity, and then blocked with 0.8% (v/v) Block Ace (DS Pharma Biomedical, Osaka, Japan) in PBST. After overnight incubation at 4°C with primary antibodies in 0.4% Block Ace/PBST, sections were further incubated with goat polyclonal anti-rabbit IgG antibody, conjugated to biotin (Vector Laboratories), followed by the avidin–biotin complex. Peroxidase activity was detected using diaminobenzidine as the chromogen. Bright-field images were acquired using the NanoZoomer Digital Pathology virtual slide scanner. In the quantitative analysis, neuroanatomic areas were determined according to a mouse brain atlas (Franklin and Paxinos, 1997), and the experimenter was blind to the experimental groups.

### Monoamine and acetylcholine measurement

Circular tissue punches (1 mm in diameter) were obtained from 150 μm-thick frozen coronal brain sections from 4 to 5-month-old mice (*n* = 8/group) and stored at −80^°^C until assayed. Monoamines and metabolites were extracted and measured using HPLC with electrochemical detection as described previously (Sano et al., 2009). Acetylcholine was measured using a combination of HPLC, enzymatic reactions, and electrochemical detection (Eicom, Kyoto, Japan), as described previously (Itoh et al., 1999).

### Behavioral testing and nicotine administration

#### 5-choice serial reaction time task (5-CSRTT)

The 5-CSRTT chamber (O'HARA & Co., Tokyo, Japan) comprises a fan-shaped arena with a curved wall containing 5 holes (1.5 cm diameter) in the front, side-walls (13.5 cm h), and a food dispenser located in the back corner, ensuring that the distances between the food dispenser and holes were roughly equal (11 cm). The holes on the curved wall were located 3 cm above the floor and at 3-cm intervals. The food comprised sweetened pellets (10 mg each, Test Diet, Richmond, IN). Infrared beam sensors detected nose-pokes and food intake. Each chamber was installed in an independent soundproof box.

Training was conducted according to a previously described procedure (Patel et al., 2006) with minor modifications. In brief, male mice were handled and food restricted to reduce their body weight to approximately 85% of free-feeding weight, habituated to the chamber for 30 min daily for 2 days, and then trained to consume food pellets from a pellet dispenser for 15 min daily for 3 days. Twenty trials were given at 45-s intervals. The mice were then trained to associate nose-pokes with food in a non-specific manner for 30 min on 3 consecutive days (100 trials/day). The intertrial interval (ITI) was 2 s, and the limited hold (LH) of the green signal lights behind all of the holes was 60 s. In the following sessions (Spatial stages 1–10), only 1 of 5 holes was illuminated in a random manner with a different ITI and LH. A single session comprised 100 trials or a maximum of 30 min. The ITI and LH were 2 s and 60 s (Spatial stage 1, 14 sessions), 10 s and 60 s (Spatial stage 2, 3 sessions), 10 s and 30 s (Spatial stage 3, 7 sessions), 10 s and 10 s (Spatial stage 4, 7 sessions), 15 s and 10 s (Spatial stage 5, 2 sessions), 20 s and 10 s (Spatial stage 6, 4 sessions), 20 s and 5 s (Spatial stage 7, 8 sessions), 20 s and 2 s (Spatial stage 8, 6 sessions), 20 s and 1 s (Spatial stage 9, 7 sessions), and 20 s and 0.8 s (Spatial stage 10, 7 sessions), respectively. In Spatial stages 2–10, the house light was extinguished for 2 s if a mouse did not perform a nose-poke within the LH (omission) or performed a nose-poke into a wrong hole (incorrect). A nose-poke before the ON signal light was considered a premature nose-poke. A premature nose-poke led to no reward, but no additional punishment was associated with it to avoid a severe decline in motivation to continue the tasks.

(-)-Nicotine hydrogen tartrate salt was dissolved in PBS and administered subcutaneously at 0, 0.35, 1.05, 3.5, 10.5, and 35 μg/kg (free base) once daily, 10 min prior to the task (conditions: 15 s ITI, 60 s LH). The same group of mice was injected in incremental steps, and each dose level was tested twice. The data obtained from the 2 days of recording were averaged.

For c-Fos analyses, the mice were trained up to the 3rd session of Spatial stage 2, and then transcardially perfused first with normal saline and then with 4% PFA under deep anesthesia 90 min after the start of the last session.

#### New environment adaptation

Mice were placed in a transparent plastic cage [46 (W) × 24 (L) × 20 (H) cm] without bedding materials for 10 min per day for 3 days and for 5 min on the 4th day. Locomotor activity was measured using infrared beam sensors (Scanet, Melquest, Toyama, Japan). Nicotine was administered subcutaneously at 35 μ g/kg (free base), once daily 10 min prior to the task.

#### Decision-making tests

An automated T-maze apparatus (O'HARA & Co., Tokyo, Japan) was used. The T-maze comprised a start arm and 2 goal arms (each 41 cm long) with V-shaped high-sided walls (floor, 3 cm w; opening, 11.5 cm w; 15.5 cm h). The start and goal boxes were attached to the arm ends. Goal boxes contained food dispensers set to deliver defined numbers of sweetened pellets (10 mg each, Test Diet). Infrared beam sensors detected food intake. Goal boxes were directly connected to the start box by corridors. Thus, mice autonomously returned to the start box after each trial. Computer controlled push-up gates (15.5 cm h) were placed at the entrances and exits of the start and goal boxes. An additional gate was placed 4 cm from the entrance of the goal arm to prevent backward movement after making a choice. All doors and walls were gray in color.

Delay-based and effort-based decision-making tests were administered according to published procedures (Rudebeck et al., 2006), with modifications for mice. In both protocols, mice were initially handled (2 min/day), food restricted to approximately 80% of free-feeding weight, and habituated to the maze, baited with scattered pellets (5 min/day), for 5 days. (*Free arm entry sessions):* Mice were allowed free access to sweetened pellets in both the high reward arm (HRA) and low reward arm (LRA) without gates or obstacles. Ten reward pellets were available in the HRA, with only one reward pellet was available in the LRA. The session consisted of 6 trials per day for 5 days.

***Delay-based decision-making test:*** *(Forced arm choice)*. Mice were forced to visit either the HRA (3 trials) or LRA (3 trials) by closing the opposite gate. The arm-entry order was semi-randomized, and a session of 6 trials per day for 5 days was undertaken. The HRA (containing 6 reward pellets) and LRA (containing 1 reward pellet) were kept constant throughout the following sessions, and counter-balanced among samples. *(Free choice training and testing sessions)* Mice were allowed to freely and without delay select either arm for 7 days until they selected the HRA in 6 of the 8 most recent trials, or a maximum of 10 trials per day. The HRA was then associated with a 5-s delay for 7 days, a 10-s delay for 5 days, and a 15-s delay for 5 days. In the 10-s and 15-s delay sessions, the trial numbers were increased to a maximum of 20 per day.

***Effort-based decision-making test:*** *(Forced arm choice)*. Mice were forced to visit either the HRA (3 trials, 6 reward pellets) or the LRA (3 trials, 1 reward pellet) by closing the opposite gate. The order of arm entry was semi-randomized, and 6 trials per day for 5 days were performed. The HRA contained a 15-cm tall obstacle (90° angle for climbing up and 45° angle for climbing down, covered with a soft mesh for gripping), while the LHA contained no obstacle. (*Free arm choice training and testing sessions):* Mice were allowed to freely select either arm until they chose the HRA in 6 of the 8 most recent trials, or for a maximum of 10 trials per day for 14 days. The HRA and LRA were kept constant throughout the sessions, and counter-balanced among samples. (*Reversal sessions):* The sides containing the HRA and LRA were reversed, and then kept constant for 14 days. The obstacle remained in the HRA (*Dual barriers sessions):* For an additional 5 days, both the LRA and HRA were presented with the same obstacle.

#### Other tests

The *Morris water maze* test was performed as described (Sano et al., 2009) with minor modifications. Mice were given 4 trials per day for 7 consecutive days under brightly illuminated conditions (200 lux at the maze surface). A probe trial was performed on day 8 after the acquisition session.

The *Open field test* was performed as described (Sano et al., 2009), with minor modifications. A white open field (50 × 50 cm) was used, and the center area was defined as the central 18 × 18 cm region of the arena.

*Home cage activity, elevated plus-maze, prepulse inhibition, eight-arm radial maze, and contextual and cued fear conditioning tests* were performed as previously described (Sano et al., 2009).

#### IntelliCage study

The IntelliCage apparatus and software (NewBehavior AG, Zurich, Switzerland, www.newbehavior.com) were described previously (Krackow et al., 2010; Voikar et al., 2010). Tasks were performed using female mice, as follows: The choice of female in Intellicage is based on their greater compatibility in a social home cage setting. Given that the estrous cycle of mice lasts 5 days, the long observation periods would most likely cancel any minor fluctuation effects.

***General procedures and adaptation phase.*** Radio frequency identification transponders (Planet ID GmbH, Essen, Germany) were implanted subcutaneously in the dorso-cervical region under isoflurane inhalation anesthesia. Thereafter, the mice were allowed to recover for 1 week, in mixed genotypes groups of 10–12 in standard Type III cages (Tecniplast, Buguggiate, Italy), with water and food available *ad libitum*. During week 1 in the IntelliCage, all doors were open, providing free access to all eight drinking bottles (free adaptation). During week 2, all doors were closed but could be opened once per visit with a nose-poke for 5 s (nose-poke adaptation). During the last week of adaptation, the mice were adapted to a fixed drinking schedule (drinking session adaptation) with doors opening in response to nose-pokes between the hours of 11:00–12:00 and 16:00–17:00 only. During all adaptation phases and tasks, the mice were fed *ad libitum* with standard mouse food (Kliba Nafag 3430; Provimi Kliba AG, Kaiseraugst, Switzerland) and kept on aspen bedding (5 × 5 × 1 mm, Tapvei OY, Kortteinen, Finland) changed every 1–3 weeks depending on the task schedule. Ambient lights were on between 20:00–08:00.

***Corner avoidance task.*** This task comprised a training trial followed by two probe trials (test and re-test). During the 24-h training trial, each mouse was assigned a target corner (avoiding the most and least visited corners during pre-training) in which nose-pokes triggered a 1-s air puff (0.8 bar) instead of opening a door. The training trial was followed by a 24-h retention interval outside the IntelliCage in a regular Type III cage, with water available only during the first 6 h. The mice were subsequently reintroduced into the IntelliCages for 5 days without air puffs, and with water available in all four corners, as during the nose-poke adaptation. The first and last 24 h of this period served as probe trials to monitor the retention and extinction of target corner avoidance. Avoidance was quantified as the percentage of correct visits with nose-pokes, minus the chance level of 25%.

***Corner preference, serial reversal, chaining, and patrolling tasks.*** In this set of tasks, water was available in only one of four corners during each drinking session. The rule predicting the rewarded corner varied between tasks. To begin, water was available in the same corner for 14 sessions (*corner preference*), followed by 14 sessions with water available in the opposite corner (*corner reversal*) and 8 sessions during which the mice had to learn a new corner during each drinking session (*serial reversal*). To prevent learning by imitation, cage mates were divided in four subgroups with different target corners. Next, the water was always delivered in the corner adjacent to the most recently visited one in which at least one nose-poke had been made, either in a clockwise or anti-clockwise direction. Each mouse was first trained for 14 sessions in one direction (*chaining acquisition*) and then 21 sessions in the opposite direction (*chaining reversal*). Finally, water was made available in the corner adjacent to the last rewarded corner and the mice were again trained for 17 sessions in one direction (*patrolling acquisition*) and then 21 sessions in the opposite direction (*patrolling reversal*). The patrolling task is more difficult to learn than the chaining task, because the target corner is not adjusted if the animal makes an error. Performance was quantified as the percentage of correct visits with nose-pokes, minus the chance level of 25%.

***Reaction time task.*** In this task, all four corners operated in the same way, 24 h per day: The first nose-poke in a visit determined the correct side and initiated a delay period, after which, on the correct side and for a period of 5 s, 3 green LEDs were switched on and the door opened for drinking. Any nose-poke during the delay period was considered a premature response, whereas the first nose-poke at the open door was counted as correct response. Correct response latency was defined as the time that elapsed between the onset of the light stimulus and the correct response. The task had three phases. During the first 3 days, delays were set at 0 s (*baseline*). Then, the delays varied randomly between 0.5, 1.5, and 2.5 s for the rest of the task. During the first 5 days, premature responses had no consequence (*training*). During the final phase of 7 days (*testing*), premature responses stopped the trial, requiring the mouse to leave the corner and to start again.

***Delay discounting task.*** In this task, all four corners operated in the same way, 24 h per day: with a given delay after onset of a visit, doors opened spontaneously for a 7-s drinking period. To force a choice of either the left or right bottle, a nose-poke at any open door closed or prevented opening of the door on the other side. The task was divided into three phases. First, with delays set at 0 s, in each corner (two left corners, two right corners), one bottle of water was replaced with 0.5% saccharin and animals were allowed to develop preferences for saccharin bottles over 5 days (*training*). In the second phase (*discounting*), delays for opening the saccharin doors were increased by 0.5 s every 24 h. After 16 days, this resulted in a delay of 8 s. For the final 4 days (*extinction*), delays were reset to 0 s. A saccharin preference score was calculated as: (lick contact time at saccharin bottles—lick contact time at water bottles)/total lick contact time. Nose-pokes at the closed saccharin door during the delay period had no consequence, but were scored as a possible measure of compulsivity.

***Statistical model for the IntelliCage study.*** In a first step, the entire analysis was run with three groups: two control groups and one mutant group. As the control groups were indistinguishable in all tests, the ANOVA was re-run with the 2 control groups merged—as presented now (mHb:DTA vs. control), between subject factors, and within subject factors to explore the dependence of genotype effects on place, time, and stimulus. Significant interactions, and where necessary, significant main effects were explored further by Tukey–Kramer *post-hoc* tests or by splitting the ANOVA model as appropriate. One-sample *t*-tests were used for follow-up comparisons against chance levels. Variables known to produce strongly skewed distributions and/or frequent outliers were subjected to log transformation before ANOVA analysis (e.g., latency measures, passive floating). The significance threshold was set at 0.05. The false discovery rate control procedure of Hochberg was applied to groups of conceptually-related variables within single tests to correct significance thresholds for multiple comparisons.

### Statistical analysis of other data

Data were analyzed using an unpaired 2-tailed *t*-test; One-Way, Two-Way, and Three-Way ANOVA; and Bonferroni and Tukey–Kramer *post-hoc* tests. Probability values less than 0.05 were considered statistically significant.

## Results

### Genetic ablation of mHb cells in mice

**Figure 1:**
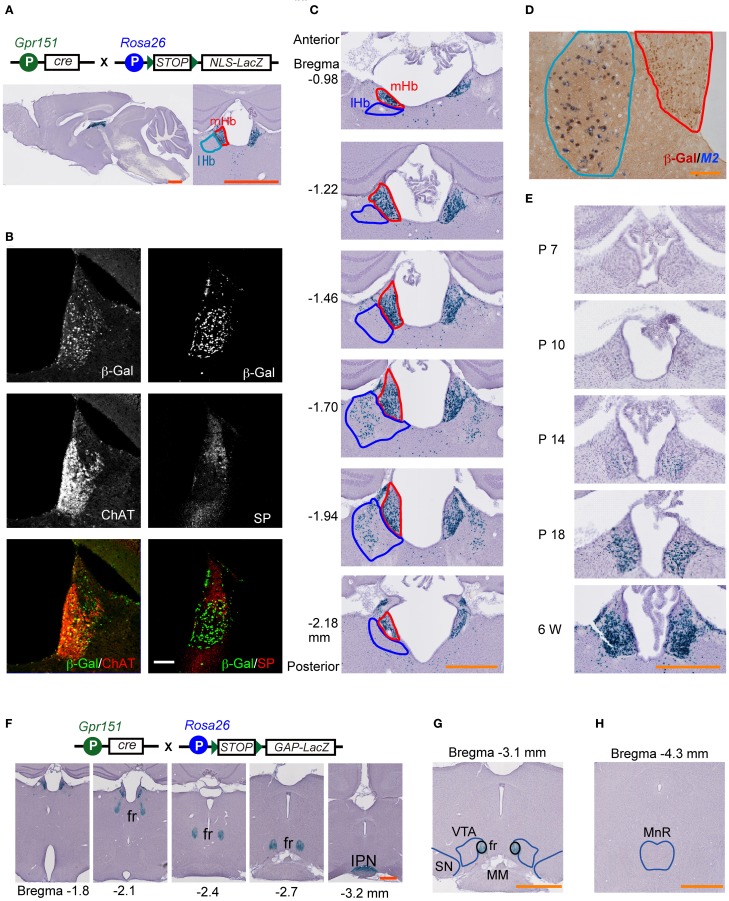
**Characterization of mHb-specific Cre expression transgenic mice.** Transgene combinations are schematically illustrated at the top of the figure **(A,F)**. **(A)** β-galactosidase (β-Gal) activity (blue signals) in *Gpr151*-Cre:RNZ mouse brain sections. The left and right panels show sagittal and coronal sections, respectively. Blue signals represent nuclei of cells expressing Cre protein under a *Gpr151* promoter. The medial habenula (mHb) and lateral habenula (lHb) are encircled by red and blue lines, respectively. **(B)** Protein localization of β-Gal, choline acetyltransferase (ChAT), and substance P (SP) in the Hb of *Gpr151*-Cre:RNZ mice. **(C)** Cre-mediated recombination occurred preferentially in the ventral area of the mHb, across the entire anteroposterior axis of the habenular nuclei. Only a small population of lHb neurons in the posterior area exhibited recombination. **(D)** The majority of cells labeled with β-Gal protein (brown) in the lHb were characterized by *Chrm2* (muscarinic acetylcholine receptor 2; M2) (blue) expression in *Gpr151*-Cre:RNZ mice. The mHb and lHb are outlined by red and blue lines, respectively. **(E)** β-Gal staining on postnatal days (P) 7, 10, 14, and 18, and 6 w. Recombination mediated by Cre protein started between P7 and P10 and progressively increased by P18. (**F–H)** LacZ staining of *Gpr151*-Cre:RGZ mice revealed β-Gal activity in the fasciculus retroflexus (fr), but not in the monoaminergic centers such as the ventral tegmental area (VTA), substantia nigra (SN), and medial raphe nucleus (MnR). **(F)** Blue signals represent axons originating from recombined cells. These cells project to the IPN through the fr. Representations of the fr, VTA, and SN **(G)**, and MnR **(H)** are indicated by blue lines. MM, mammillary nucleus. Scale bars = 1 mm **(A,G,H)**, 500 μm **(C,E,F)**, 100 μm **(B)**, and 50 μm **(D)**.

We generated Cre-recombinase transgenic mouse lines using a bacterial artificial chromosome clone containing *Gpr151 (GalRL*, *GPCR2037*, and *PGR7)*. After crossing the created line with *Rosa26-STOP-NLSLacZ* (RNZ) reporter mice, the recombination specificity of multiple lines was characterized, and a representative line chosen (hereafter referred to as *Gpr151*-Cre). The NLS helps to constrain β-Gal within the cell nucleus, allowing for identification at single-cell resolution. Double heterozygous (*Gpr151*-Cre:RNZ) mice exhibited β-Gal activity preferentially in ventral mHb cells (Figures 1A–C). Immunohistochemistry revealed that more than 57% of ChAT-positive mHb cells were also β-Gal-positive (Figure 1B), indicating that the targeted cells possessed cholinergic characteristics. The β-Gal and SP immunoreactivity overlapped slightly in the dorsal area of the mHb (Figure 1B). We detected β-Gal activity in a limited population of cells in the posterior but not anterior area of the lHb (Figure 1C). This minor subset of cells in the lHb was characterized by the expression of muscarinic acetylcholine type 2 receptors (Figure 1D). Small fractions of cells scattered in the paraventricular (PVT) and reuniens thalamic nuclei (ReT) were β-Gal-positive at approximately 5% and 7%, respectively. No other brain regions exhibited β-Gal activity. Thus, the vast majority of Cre-mediated recombination was found in the mHb cholinergic neurons. Ontogenic studies revealed β-Gal activity in the mHb by postnatal day 10 but not by day 7, and this activity increased progressively to postnatal day 18, indicating postnatal onset of Cre-mediated recombination in *Gpr151*-Cre mice (Figure 1E). Cre-mediated recombination was saturated by early adulthood (6 weeks old) (Figure 1E).

To visualize the projection areas of the recombinant cells, we crossed *Gpr151*-Cre mice with Rosa26-STOP-GAPLacZ (RGZ) reporter mice. The palmitoylation signal sequence of GAP43 facilitates β-Gal distribution along axonal projections. Double transgenic *Gpr151*-Cre:RGZ mice displayed β-Gal signals in mHb, the fasciculus retroflexus, and IPN (Figure 1F). β-Gal signals were not observed in the monoaminergic centers (Figures 1G,H), which are directly innervated by lHb neurons. These results indicate that *Gpr151*-Cre preferentially targeted mHb cells projecting to the IPN.

**Figure 2:**
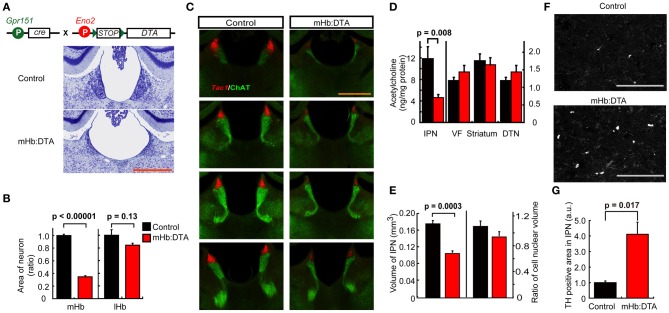
**mHb-selective lesions in mHb:DTA mice. (A)** Neurons visualized by Nissl staining in the mHb and lHb of control and mHb:DTA mice. **(B)** Relative areas of Nissl stained neurons in the mHb and lHb of control and mHb:DTA mice (mice were 7–8 months old, *n* = 5/group, *t*-test). **(C)** Expression pattern of *Tac1* (tachykinin 1, substance P) mRNA (red) and ChAT protein (green) in control and mHb:DTA mice at 3–4 months. ChAT localized to the cell bodies of the ventral area of the mHb and fr. *Tac1* and ChAT signals were diminished in mHb:DTA mice, suggesting local interaction between subsets of mHb neurons. **(D)** Levels of acetylcholine in the IPN, ventral forebrain (VF), striatum, and dorsal tegmental nucleus (DTN; mice were 4–5 months old, *n* = 8/group, *t*-test). The left *y*-axis scale relates to the IPN and the right *y*-axis scale relates to other regions. **(E)** Quantitative analysis of the IPN area. Areas of the IPN, delineated by strong tyrosine hydroxylase (TH) immunostaining in surrounding tissue in sequential sections, were summed. The left *y*-axis indicates the volume of the IPN and the right *y*-axis represents the ratio of cell nuclear volume. The IPN volume was significantly smaller in mHb:DTA mice than in controls, while there were no differences between genotypes in the cell nuclear areas revealed by DAPI (4′,6-diamidino-2-phenylindole, dihydrochloride) signals. **(F)** Confocal images of TH-positive puncta in the IPN. There were more detectable puncta in mHb:DTA mice. **(G)** Relative values showing the areas of TH-positive puncta in the IPN of control and mHb:DTA mice, normalized to 1.0 for control mice. TH-positive puncta were significantly larger and higher in number in mHb:DTA mice than in controls, indicating enhanced sprouting of axon terminals from the locus coeruleus (LC; mice were 7–8 months old, *n* = 3/group, *t*-test). a.u., arbitrary unit. Data are shown normalized to 1.0 for the control mice. Scale bars indicate 500 μm **(A,C)** and 75 μm **(F)**. Data represent mean ± standard error of the mean (SEM).

We then crossed *Gpr151*-Cre mice with *Eno2*-STOP-DTA mice (Kobayakawa et al., 2007). Cre-mediated recombination deletes STOP sequences and allows for expression of the DTA subunit under control of the *Eno2* promoter, leading to the death of Cre-expressing neurons (Figures 2A,B). We analyzed the relative number of neurons in the mHb and lHb by measuring the areas of Nissl-positive staining. The neuronal area in *Gpr151*-Cre:*Eno2*-STOP-DTA (hereafter mHb:DTA) mice showed a significant decrease (~65%) compared with that of control mice (Figure 2B). It is interesting to note that SP-nergic and cholinergic cells were substantially reduced in the mHb (Figure 2C), suggesting a role for intra-mHb communication in the survival of SP-nergic cells. As a result, the transverse area of the fasciculus retroflexus was 58% in control mice. In contrast, in the lHb, the cell numbers did not significantly decrease relative to controls (Figure 2B). Furthermore, cell densities in the PVT and ReT of mHb:DTA mice were not significantly different from those in control mice [87.2 ± 5.4% (*p* = 0.09, *n* = 16 slices/genotype, *t*-test) and 94.8 ± 4.5% (*p* = 0.4, *n* = 16 slices/genotype, *t*-test), respectively]. To determine the efficiency of Cre/DTA-mediated cell ablation, we examined β-Gal activity in *Gpr151*-Cre:RNZ double-transgenic and Gpr151-Cre:RNZ:DTA triple-transgenic mice at P16 and in early adulthood (6 weeks old). β-Gal activity was not detected in *Gpr151*-Cre:RNZ:DTA triple-transgenic mice at either stage, indicating efficient cell death induced by DTA (data not shown).

To assess the effect of mHb cell ablation on the IPN, we analyzed the IPN neurochemically and histologically. The acetylcholine concentration was decreased by 60% in the IPN of mHb:DTA mice but remained unchanged in all other brain areas examined (Figure 2D). The IPN volume in mHb:DTA mice was reduced by approximately 40% compared with that of controls, although cell nuclear staining revealed no evidence of cell loss (Figure 2E), suggesting axonal and/or dendritic loss in this area. Furthermore, TH-immunoreactive puncta were increased in the IPN (Figures 2F,G). Consistent with the histochemical data, we detected a selective and significant increase in noradrenaline in IPN punch samples (Table 1, *p* < 0.05, two-tailed *t*-test). Because locus coeruleus (LC) neurons project to the IPN and release noradrenaline, these results suggest that LC neurons exhibit compensatory responses (Battisti et al., 1987). We did not detect differences in the levels of any other monoamines or differences in any other examined brain areas, such as the ventral forebrain, VTA, raphe nucleus, and striatum (Table 1). We observed no sex differences in the neurochemical and histochemical characteristics of mHb:DTA mice, and thus data from both males and females were pooled for analyses. Taken together, these data demonstrate selective disruption of the mHb–IPN pathway in mHb:DTA mice.

**Table 1:** **Levels of monoamines and metabolites in selected brain regions**.

**Region**	**Genotype**	**NA**	**MHPG**	**DA**	**DOPAC**	**HVA**	**5-HT**	**5-HIAA**
IPN	Control	4043 ± 109	n.d.	1738 ± 239	1654 ± 211	2111 ± 115	3561 ± 214	6589 ± 429
	mHb:DTA	5169 ± 384	n.d.	2513 ± 331	2182 ± 300	2424 ± 212	3829 ± 150	7014 ± 410
	*p* value	**0.030**		0.101	0.205	0.263	0.312	0.494
Raphe	Control	8664 ± 579	n.d.	1540 ± 178	652 ± 84	1283 ± 93	9864 ± 438	9342 ± 526
	mHb:DTA	8923 ± 584	n.d.	1513 ± 158	599 ± 39	1224 ± 60	10,121 ± 399	9685 ± 378
	*p* value	0.763		0.913	0.548	0.589	0.674	0.595
VTA	Control	4976 ± 329	n.d.	4305 ± 657	3200 ± 322	3334 ± 203	4477 ± 515	7501 ± 308
	mHb:DTA	4701 ± 265	n.d.	3546 ± 441	2975 ± 237	3223 ± 214	3947 ± 368	7615 ± 366
	*p* value	0.523		0.338	0.575	0.720	0.405	0.825
mPFC	Control	2980 ± 289	ND	191 ± 25	318 ± 43	791 ± 46	1096 ± 106	2729 ± 127
	mHb:DTA	2792 ± 71	ND	200 ± 23	280 ± 10	728 ± 47	1102 ± 34	2547 ± 69
	*p* value	0.511		0.785	0.376	0.358	0.953	0.213
Striatum	Control	ND	2273 ± 336	66,198 ± 5503	14,616 ± 1356	9707 ± 840	1638 ± 135	1852 ± 163
	mHb:DTA	ND	2568 ± 278	69,070 ± 6685	13,244 ± 1201	9671 ± 704	1627 ± 140	1833 ± 165
	*p* value		0.509	0.745	0.461	0.975	0.958	0.937

*Data are shown as mean ± SEM (pg/mg protein). Two tailed T-test for statistics. n.d., not detected; ND, not determined; 5-HT, 5-hydroxytryptamine; 5-HIAA, 5-hydroxyindoleacetic acid; NA, noradrenaline; MHPG, 3-methoxy-4-hydroxy-phenylglycol; DA, dopamine; DOPAC, 3,4-dihydroxyphenylacetic acid; HVA, 3-methoxy-4-hydroxyphenylacetic acid (homovanillic acid)*.

### Behavioral phenotypes of mHb:DTA mice

**Figure 3:**
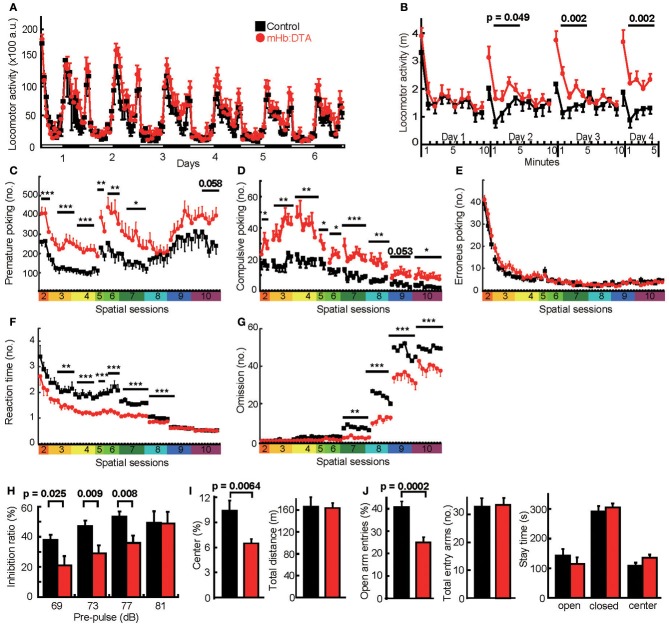
**Maladaptation and impulsive/compulsive behavior in mHb:DTA mice. (A)** Locomotor activity of control (black) and mHb:DTA (red) mice in home cages (mice were 5–6 months old, *n* = 12/group). Levels of locomotor activity per hour. White and black bars represent light (day) and dark (night), respectively. **(B)** mHb:DTA mice failed to show habituation during repeated exposure to a new environment [mice were 7–8 months old, *n* = 12/group; for days 1, 2, 3, and 4; genotype, *F*_(1, 22)_ = 0.47, 4.36, 12.5, and 12.3, respectively; genotype × time, *F*_(1, 22)_ = 0.90, 1.15, 3.95, and 1.49, *P* = 0.46, 0.33, 0.0053, and 0.21, respectively]. (**C–G)** 5-CSRTT (mice were 6–8 months old, *n* = 12/group). **(C)** Total number of premature nose-pokes was significantly higher in mHb:DTA mice [spatial sessions 2, 3, 4, 5, 6, and 7: *F*_(1, 22)_ = 17.2, 14.6, 16.9, 8.16, 8.85, and 5.30, respectively, genotype × day interaction, *P* > 0.05 for all sessions]. **(D)** Compulsive nose-pokes after success was increased in mHb:DTA mice [spatial sessions 2–10: genotype effect, *F*_(1, 22)_ = 4.90, 12.9, 18.8, 4.68, 6.65, 15.6, 10.1, and 6.47, respectively, genotype × day interaction, *P* > 0.05 for all sessions]. **(E)** The number of erroneous nose-pokes was comparable between mHb:DTA mice and control mice. **(F)** Latency to poke the correct hole was shorter in mHb:DTA mice [spatial sessions 2–10: genotype, *F*_(1, 22)_ = 2.76, 9.53, 24.67, 21.81, 13.87, 46.64, 35.23, 1.46, and 0.034, respectively, genotype × day/session interaction, *P* > 0.05 for all sessions except for *P* = 0.021 for session 8]. **(G)** The number of omissions was decreased in mHb:DTA mice [spatial sessions 2–10; genotype, *F*_(1, 22)_ = 2.20, 1.71, 2.79, 0.41, 2.77, 9.62, 33.83, 16.32, and 9.35, respectively; genotype × day/session interaction, *P* > 0.05 for all sessions except for *P* = 0.019 for session 8]. **(H)** Reduced inhibition ratio in an auditory prepulse inhibition test [mice were 4–5 months old, *n* = 12/group, Two-Way ANOVA, genotype × prepulse interaction, *F*_(3, 95)_ = 3, *P* = 0.036, shown are Bonferroni *post-hoc* tests]. Background white noise was given at 65 dB. **(I)** Ratio of time spent in the center area (left panel) and total distance traveled (right panel) in the new open field (50 × 50 cm) test (mice were 5–6 months old, *n* = 12/group, *t*-test).** (J)** Ratio of open arm choice (left panel), number of total entry arms (middle panel), and time spent in open and closed arms and center area (right panel) in the elevated plus-maze test (mice were 5–6 months old, *n* = 12/group, *t*-test). Data represent mean ± SEM. a.u., arbitrary unit; ^*^p < 0.05; ^**^p < 0.01; and ^***^p < 0.001.

To determine the role of the mHb–IPN pathway, we performed extensive behavioral analyses. To avoid the effects of estrous cycles, we used males in all of the conventional behavioral tests. The mHb:DTA mice did not differ from control mice in home cage activity, although they tended to be hyperactive during the early night period (Figure 3A). We also observed no difference in locomotion between control and mHb:DTA mice when they were initially exposed to new environments (Figure 3B). In control mice, repeated daily exposure to a new environment caused typical habituation. Interestingly, the mHb:DTA mice showed no significant habituation. This was evident during the first 5 min of exposure to the new environment [days 2, 3, and 4; genotype, *F*_(1, 22)_ = 4.36, 12.5 and 12.3, *P* = 0.049, 0.002, and 0.002, respectively; time, *P* < 0.0001 for all days; genotype × time interaction, *P* > 0.05 for all days, repeated measures ANOVA (rmANOVA); Figure 3B], suggesting deficits in acquiring and/or evaluating environmental information. These phenotypes were highly reproducible in independent animal groups, as described in later sections.

The 5-CSRTT is used to evaluate both impulse control and attention in rodents (Robbins, 2002; Patel et al., 2006). In this test, we observed a profound increase in premature responses in mHb:DTA mice, from the early behavioral shaping stages to the later attention-testing stages (Figure 3C), indicating impulsive behavior. Differences were particularly evident in spatial sessions 2–7, with a similar tendency in later sessions [spatial sessions 2–10; genotype, *F*_(1, 22)_ = 17.16, 14.61, 16.94, 8.16, 8.85, 5.30, 0.61, 1.58, and 3.99, *P* = 0.0004, 0.0009, 0.0005, 0.009, 0.007, 0.031, 0.442, 0.222, and 0.058, respectively; genotype × day/session interaction, *P* > 0.05 for all sessions; rmANOVA]. We also observed a higher rate of perseverative nose-pokes within 5 s after correct responses [spatial sessions 2–10; genotype, *F*_(1, 22)_ = 4.91, 12.94, 14.78, 4.69, 6.65, 15.64, 10.13, 4.16, and 6.47, *P* = 0.037, 0.0016, 0.0003, 0.042, 0.017, 0.0007, 0.0043, 0.054, and 0.019, respectively; genotype × day/session interaction, *P* > 0.05 for all sessions; rmANOVA; Figure 3D], probably reflecting compulsiveness, because it would take a mouse longer than 5 s to consume a 10-mg pellet. These phenotypes were also highly reproducible in independent groups of animals, as described later. We observed no difference in the erroneous nose-pokes, suggesting unaltered spatial attention (spatial sessions 2–10; genotype, *P* > 0.05 for all sessions, rmANOVA; Figure 3E). On the other hand, the latency to poke the correct hole was significantly shorter in mHb:DTA mice than in controls [spatial sessions 2–10; genotype, *F*_(1, 22)_ = 2.76, 9.53, 24.67, 21.81, 13.87, 46.64, 35.23, 1.46, and 0.034, *P* = 0.11, 0.0054, 0.0001, 0.0001, 0.0012, 0.0001, 0.0000, 0.23 and 0.855, respectively; genotype × day/session interaction, *P* > 0.05 for all sessions except for spatial 8, *P* = 0.021; rmANOVA; Figure 3F] and the number of omissions was significantly reduced in mHb:DTA mice [spatial sessions 2–10; genotype, *F*_(1, 22)_= 2.20, 1.71, 2.79, 0.41, 2.77, 9.62, 33.83, 16.32, and 9.35, *P* = 0.15, 0.20, 0.11, 0.52, 0.11, 0.0052, 0.0000, 0.0005 and 0.0058, respectively; genotype × day/session interaction, *P* > 0.05 for all sessions except for spatial 8, *P* = 0.019; rmANOVA; Figure 3G]. These data suggested that mHb:DTA mice more rapidly reacted to target stimuli in accordance with high impulsivity without deficits in attention performance. In a prepulse inhibition test, mHb:DTA mice exhibited impaired acoustic prepulse inhibition [Two-Way ANOVA, genotype × prepulse interaction, *F*_(3, 95)_ = 3, *P* = 0.036, Bonferroni *post-hoc* test, *P* = 0.025, 0.009, 0.008 and 0.96 for pre-pulse 69, 73, 77, and 81 dB; Figure 3H] with normal startle responses, however, which represents another form of attention.

We observed a modest increase in the anxiety levels of mHb:DTA mice, indicated by a decrease in the frequency of visits to the center area of the open field (*P* = 0.0064, two-tailed *t*-test; Figure 3I) and to the open arms of the elevated plus maze (*P* = 0.0002, two-tailed *t*-test; Figure 3J), with no differences in total locomotor activity during the observation period in either test. The genotypes exhibited no differences, however, in time spent in the open and closed arms and the center area of the elevated plus maze (Figure 3H, right panel). The data shown were obtained from the same groups of mice that underwent the tests in the following sequence: open field followed by elevated plus maze. Additional independent groups of mice performed similarly. These results suggest that the lesion has a modest impact on anxiety-related behaviors.

**Figure 4:**
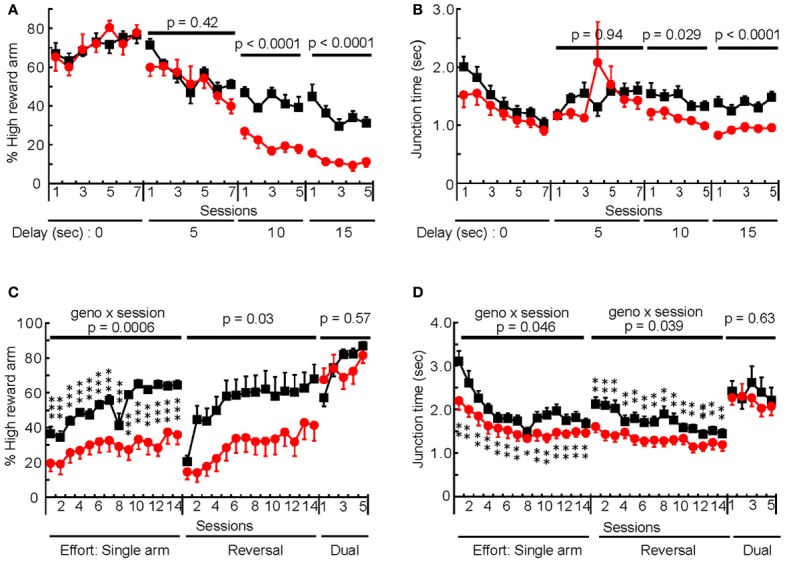
**Delay-based and effort-based decision-making in mHb:DTA mice. (A,B)** Delay-based decision-making test in mHb:DTA mice (mice were 12–14 months-old, control *n* = 10, mHb-DTA *n* = 8). **(A)** The percentage of high reward arm choice was significantly decreased in mHb:DTA mice when the delay period was 10 s or longer [Delay periods 5, 10, and 15 s: *F*_(1, 16)_ = 0.681, 32.4, and 31.6, respectively; genotype × day interaction, *P* > 0.05 for all sessions]. **(B)** Mean time spent in the junction area of the T-maze was significantly shorter in mHb:Cre:DTA mice when the delay period was 10 s or longer. [Delay period 5, 10, and 15 s: *F*_(1, 16)_ = 0.00632, 5.77, and 33.2, respectively genotype × day interaction, *P* > 0.05 for all sessions]. **(C,D)** Effort-based decision-making test in mHb:DTA mice (mice were 12–14 months-old, *n* = 11/genotype). **(C)** The percentage of high reward arm choice was significantly decreased in mHb:DTA mice when an obstacle was placed in this arm. mHb:DTA mice chose the high reward arm when obstacles were placed in both goal arms [Session Single arm, Reversal, and Dual arms: *F*_(1, 20)_ = 20.4, 5.48, and 0.328, respectively]. **(D)** Mean time spent in the junction of the two goal arms was significantly shorter in mHb:Cre:DTA mice if the obstacle was placed only in the high reward arm [Session Single arm, Reversal, and Dual arms: *F*_(1, 20)_ = 4.83, 6.67, and 0.236, respectively]. Data represent mean ± SEM. *Post-hoc* Tukey–Kramer; ^*^p < 0.05; ^**^p < 0.01; and ^***^p < 0.001.

Impulsive and compulsive behaviors have a multidimensional nature (Fineberg et al., 2010). To gain further insight into the impulsive/compulsive behaviors exhibited by mHb-DTA mice, we examined the effects of delay and effort on their decision-making behavior, using a T-maze. The mHb-DTA mice visited the LRA more frequently than control mice if the delay was 10 s or longer [delay 5, 10, and 15 s; genotype, *F*_(1, 16)_ = 0.68, 32.4, and 31.6, *P* = 0.42, < 0.0001, and <0.0001, respectively; genotype × session interaction, *P* > 0.05 for all delays, rmANOVA; Figure 4A]. The results clearly showed that mHb-DTA mice discounted the reward values by time. Under these conditions, mHb-DTA mice moved past the junction zone of the T-maze faster, representing an impulsive choice [delay 10 and 15 s; genotype, *F*_(1, 16)_ = 5.77 and 33.2, *P* = 0.029 and <0.0001, respectively; genotype × session interaction, *P* > 0.5 for both, rmANOVA; Figure 4B]. Interestingly, mHb-DTA mice visited the LRA more frequently if the HRA was associated with effort [such as climbing the obstacle; genotype, *F*_(1, 20)_ = 20.36 and 5.48, *P* = 0.0002 and 0.03; genotype × session interaction, *P* = 0.0006 and 0.17, rmANOVA: Figure 4C], while the control mice had a consistent preference for the HRA. Importantly, the mHb-DTA and control mice preferred to visit the HRA if both arms were equally equipped with obstacles [genotype, *F*_(1, 20)_ = 0.33, *P* = 0.57, rmANOVA; Figure 4C: Dual efforts]. The data clearly indicated that the mHb-DTA mice recognized reward value. The mHb-DTA mice passed the junction zone faster if an obstacle was placed unilaterally [genotype, *F*_(1, 20)_ = 4.83 and 6.67, *P* = 0.04 and 0.018; genotype × session interaction, *P* = 0.046 and 0.039, rmANOVA; Figure 4D]. Both mHb-DTA and control mice increased their stay time in the junction area, and there was no difference between the genotypes if both choices involved equal effort [genotype, *F*_(1, 20)_ = 0.24, *P* = 0.63, rmANOVA; Figure 4D: Dual efforts]. These results indicate that mHb-DTA mice, like control mice, value the reward, but for mHb-DTA mice, the reward is strongly devalued by delay and effort.

**Figure 5:**
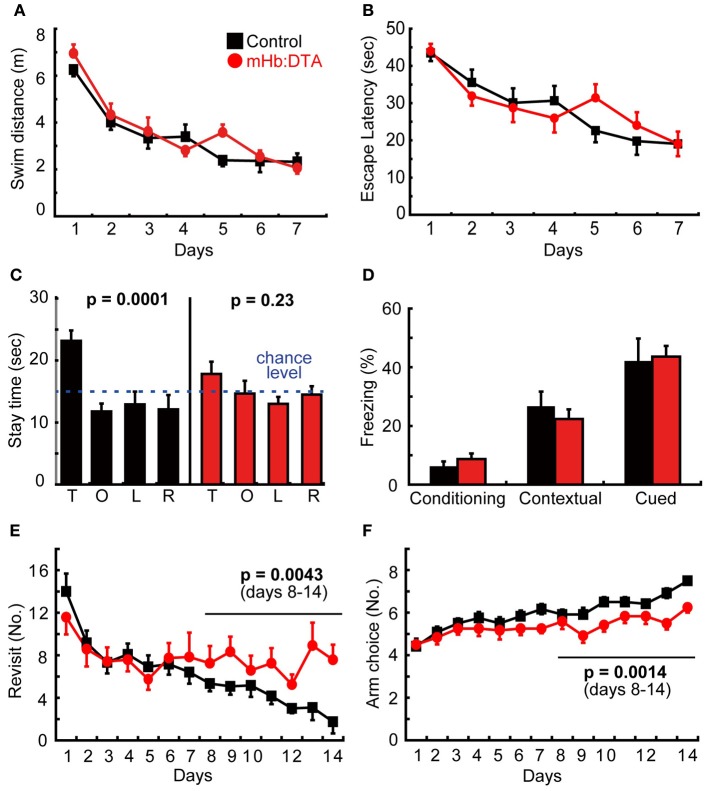
**Learning and memory in mHb:DTA mice. (A–C)** Spatial learning and memory in the Morris water maze (mice were 7–8 months-old, *n* = 12/group). **(A)** There were no differences between genotypes in the acquisition rates, swim distances **(A)**, or escape latency **(B)**. **(C)** Probe tests revealed significant quadrant preference for the controls [*F*_(3, 44)_ = 8.5] but not mHb:DTA mice [*F*_(3, 44)_ = 1.5]. T, O, L, and R represent target, opposite, left and right quadrants, respectively. **(D)** There were no differences between genotypes in freezing rates during conditioning or contextual- and cue-dependent memory tests (mice were 6–7 months old, *n* = 12/group). **(E,F)** Radial maze test. Number of revisit errors **(E)** and arms chosen during the first 8 visits **(F)** (mice were 3–4 months old; *n* = 12/group). Statistical analyses were performed for data from days 8 to 14, *F*_(1, 22)_ = 10.1 **(E)** and 13.4 **(F)**. Data represent mean ± SEM.

In the Morris water maze, mHb:DTA mice exhibited no differences in acquisition rate, represented by the swim distance and latency to reach the platform (Figures 5A,B), but showed poor spatial memory in the probe test (*P* = 0.0001 for control and 0.23 for mHb:DTA, One-Way ANOVA; Figure 5C). It should be noted that mHb:DTA mice behaved normally in the visible version of the water maze (data not shown). The data shown in Figures 5A–C are from one test. Two additional tests using independent animal groups confirmed the deficits in spatial memory revealed by the probe tests. In one test, mHb:DTA mice took longer to reach the platform, though the swim distance did not differ from that of the control mice, which reflects a longer floating time. In the last test, mHb:DTA mice showed slower learning curves in both escape latency and swim distance. Thus, we observed some variability in learning phases, and consistently observed spatial memory deficits in the mHb:DTA mice. The deficits may be due to mechanisms underlying the maintenance and/or retrieval of memory rather than acquisition. In the fear conditioning tests, we observed no differences between genotypes at any stage of conditioning, or in context-dependent and cue-dependent memory testing (Figure 5D). The data were also reproduced in independent groups of animals. Finally, we examined spatial working memory with a radial arm maze. Representative data from Test 1 are shown. The mHb-DTA mice made more revisits to arms that had been visited previously [days 8–14; genotype, *F*_(1, 22)_ = 10.21, *P* = 0.0043; genotype × day interaction, *P* = 0.2, rmANOVA; Figure 5E], and fewer visits to new arms within the initial 8 choices [days 8–14; genotype, *F*_(1, 22)_ = 13.39, *P* = 0.0014; genotype × day interaction, *P* = 0.24, rmANOVA; Figure 5F]. In two additional tests, mHb-DTA mice made consistently fewer new arm visits in the first 8 choices compared with control mice [Test 2, days 8–14, genotype, *F*_(1, 22)_ = 5.97, *P* = 0.023, genotype × day interaction, *F*_(6, 144)_ = 0.91, *P* = 0.49; Test 3, days 8–14, genotype, *F*_(1, 22)_ = 4.83, *P* = 0.038, genotype × day interaction, *F*_(6, 144)_ = 0.56, *P* = 0.76], while the tendency for revisits remained high [Test 2, days 8–14, *F*_(1, 22)_ = 2.39, *P* = 0.13; Test 3, days 8–14, *F*_(1, 22)_ = 2.69, *P* = 0.11]. Thus, we observed highly variable results in the radial arm maze tests, with a tendency that suggested working memory deficits.

### Behavior in IntelliCages

Data from the conventional behavioral tests indicated various abnormalities in mHb:DTA mice. Some behavioral phenotypes, however, may be distorted by handling and/or social isolation during these tests, which could account for the variability in the radial maze tests. Alternatively, high impulsivity may skew the behavioral data from experiments such as the Morris water maze and radial maze, which require great effort and attention for the mouse. To address this concern and/or to strengthen the behavioral data obtained from conventional tests, we used the IntelliCage system for further analyses of the mice. This system allows for fully automated and continuous testing of various behaviors under social housing conditions in a home cage environment, without the need to handle the mice (Krackow et al., 2010; Voikar et al., 2010). For the IntelliCage study, we used females to avoid potential aggression among group-housed mice. Because of long-term repetitive measurements, the estrous cycle would have little impact on the study.

**Figure 6:**
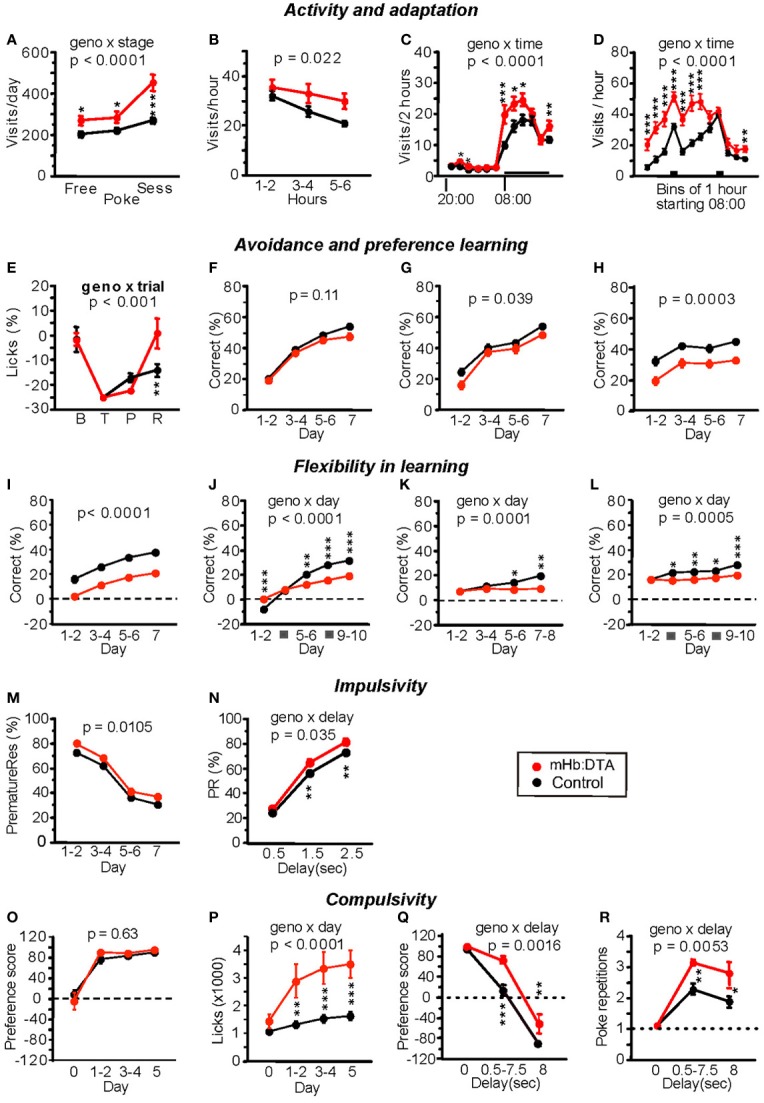
**Behavioral phenotypes of mHb:DTA mice in the IntelliCage system. (A–D)** Activity and adaptation. **(A)** Total mean visits per day during the second to last day of free, nose-poke, and session adaptation (mice were 4–5 months old, *n* = 12 for mHb:DTA and 24 for controls). The mHb:DTA mice were hyperactive during all stages, most strikingly during session adaptation [genotype *F*_(1, 33)_ = 17.3, *p* < 0.0002; stage *F*_(2, 66)_ = 51.2, *p* < 0.0001; stage × genotype *F*_(2, 66)_ = 12.7]. **(B)** Visits during the first 8 h of free adaptation. mHb:DTA mice made significantly more visits with a time curve suggesting reduced habituation [*F*_(1, 34)_ = 5.75]. **(C)** Diurnal distribution of total visits in bins of 2 h, averaged over days 2–7 of free adaptation. Horizontal bar indicates dark phase (08:00–20:00). mHb:DTA mice were hyperactive mainly during the first half of the dark phase [genotype *F*_(1, 34)_ = 6.54, *p* = 0.015; time *F*_(11, 374)_ = 133.4, *p* < 0.0001; time × genotype *F*_(11, 374)_ = 6.5]. **(D)** Distribution of visits during the dark phase in 1-h bins averaged over days 2–7 of session adaptation. Bars indicate drinking sessions (11:00–12:00, 16:00–17:00) [genotype *F*_(1, 33)_ = 31.2, *p* < 0.0001; time *F*_(11, 363)_ = 64.8, *p* < 0.0001; time × genotype *F*_(11, 363)_ = 10.0]. **(E)** Corner avoidance task. Licks to punished corner, expressed as a percentage of total licks, minus the chance level of 25%. Each point represents data for 24 h: baseline (B), training (T), probe trial (P), and retest after 3 days of extinction (R). Although mHb:DTA mice tended to show stronger avoidance of the punished corner during the probe trial, they showed an extinguished avoidance response after 3 days, unlike controls, [genotype *F*_(1, 33)_ = 0.89, ns, trial *F*_(3, 96)_ = 37.2 *p* < 0.0001, trial × genotype *F*_(3, 96)_ = 5.93]. **(F–H)** Corner preference learning. **(F)** Correct choices were not significantly affected [*F*_(1, 32)_ = 2.63]. **(G)** During reversal training, correct choices were slightly reduced in mHb:DTA mice [genotype *F*_(1, 32)_ = 5.24, genotype × day *F*_(3, 96)_ = 1.36, *p* = 0.26]. **(H)** During serial reversal training, mHb:DTA mice made fewer correct choices [genotype *F*_(1, 32)_ = 16.2, genotype × day *F*_(3, 96)_ = 0.22, *p* = 0.88]. **(I,J)** Chaining task (reward anti- or clockwise relative to last visit). **(I)** mHb:DTA mice performed less well, but learned at a similar rate as controls [genotype *F*_(1, 32)_ = 38.9, genotype × day *F*_(3, 96)_ = 0.33, *p* = 0.8]. **(J)** mHb:DTA mice learned at a slower rate but had an initial advantage over controls on the first day of reversal learning [genotype *F*_(1, 32)_ = 6.7, *p* = 0.014, genotype × day *F_(4, 128)_* = 23.8, *p* < 0.0001]. **(K,L)** Patrolling task (reward anti- or clockwise relative to last correct visit). **(K)** Patrolling acquisition: mHb:DTA mice failed to learn this task [genotype *F*_(1, 32)_ = 7.54, *p* = 0.0098, genotype × day *F*_(3, 96)_ = 7.5]. **(L)** Patrolling reversal: mHb:DTA mice failed to show learning [genotype *F*_(1, 32)_ = 7.84, *p* = 0.0086, genotype × day *F*_(4, 128)_ = 5.38]. **(M,N)** Reaction time test, testing phase. mHb:DTA mice made more premature responses and their impairment was clearly delay-dependent [**M,** *F*_(1, 31)_ = 7.42, genotype × day *F*_(3.93)_ = 0.18, *p* = 0.55; **N,** genotype *F*_(1, 31)_ = 7.432, *p* = 0.0104, delay *F*_(2, 62)_ = 1009.4, *p* < 0.0001, delay × genotype *F*_(2, 62)_ = 3.54]. **(O,P)** Saccharin preference. Day 0, water in all bottles; days 1–5, choice between water and saccharin in each corner. **(O)** Saccharin preference expressed as (S − W)/(S + W) × 100% (S = lick contact time at saccharin bottles, W = lick contact time at water bottles) rapidly reached near maximum values independent of genotype [*F*_(1, 31)_ = 0.24]. **(P)** Increase in the lick contact time in mHb-Cre:DTA mice was boosted by saccharin exposure [genotype *F*_(1, 31)_ = 15.4, *p* = 0.0004, genotype × day *F*_(3, 93)_ = 12.1]. **(Q,R)** Delay discounting: saccharin delay increased 0.5 s/24 h from 0 to 8 s. mHb:DTA mice were significantly more resistant to abandoning saccharin [**Q**, genotype *F*_(1, 31)_ = 16.1, *p* < 0.0004, delay *F*_(2, 62)_ = 273.9, *p* < 0.0001, genotype × delay *F*_(2, 62)_ = 7.17] and showed more compulsive nose-pokes at the closed saccharin door than controls [**R**, genotype *F*_(1, 31)_ = 9.9, *p* < 0.0036, delay *F*_(2, 62)_ = 58.7 *p* < 0.0001, genotype × delay *F*_(2, 62)_ = 5.71]. Data represent mean ± SEM. Tukey–Kramer *post-hoc* test; ^*^p < 0.05; ^**^p < 0.01; and ^***^p < 0.001.

Free, nose-poke, and drinking session adaptation tests revealed that female mHb:DTA mice paid more visits without nose-pokes, beginning at the very first phases and during the dark phases of all adaptation stages [genotype, *F*_(1, 33)_ = 17.34, *P* = 0.0002; genotype × stage, *F*_(2, 66)_ = 12.65, *P* < 0.0001, rmANOVA; Figure 6A]. During the first 6 h of free adaptation, mHb:DTA mice made significantly more visits to corners, with a time curve indicating abnormal habituation [genotype, *F*_(1, 34)_ = 5.75, *P* = 0.022; genotype × time, ns; rmANOVA; Figure 6B]. In addition, mHb:DTA mice were most strongly hyperactive during session adaptation (*P* < 0.0001, *post-hoc* Tukey-Kramer-test; Figure 6A) and during the first half of the dark period (genotype × time, *P* < 0.0001, rmANOVA; Figures 6C,D). Visit hyperactivity was also observed in all subsequent learning tasks.

Several tests were conducted to evaluate spatial learning. In the corner avoidance test, mHb:DTA mice learned normally, but exhibited more rapid restoration of licking in the air puff-punished corner during extinction [genotype × trial, *F*_(3, 96)_ = 5.93, *P* < 0.001, rmANOVA; *P* < 0.001, *post-hoc* Tukey-Kramer-test; Figure 6E]. In the corner preference test, mHb:DTA mice performed normally during acquisition [genotype, *F*_(1, 32)_ = 2.63, *P* = 0.11, rmANOVA; Figure 6F], but were impaired during reversal [opposite corner correct; genotype, *F*_(1, 32)_ = 4.61, *P* = 0.039; genotype × day, *P* = 0.26, rmANOVA; Figure 6G] and even more so during serial reversal [correct corner changes every session; genotype, *F*_(1, 32)_ = 16.24, *P* = 0.0003; genotype × day, *P* = 0.88, rmANOVA; Figure 6H], perseverating on previous targets. Moreover, mHb:DTA mice were strongly impaired in the chaining task [reward anti- or clockwise relative to the most recent visit; genotype, *F*_(1, 32)_ = 38.9, *P* < 0.0001; genotype × day, *P* = 0.8, rmANOVA; Figure 6I], and failed to learn the more difficult patrolling task [reward anti- or clockwise relative to last correct visit, genotype × day, *F*_(3, 96)_ = 7.51, *P* = 0.0001; rmANOVA; *P* < 0.05 or 0.01 at days 5–8, *post-hoc* Tukey-Kramer-test; Figure 6K]. The deficits were more prominent in reversal tests in both the chaining task [genotype × day, *F*_(4, 128)_ = 23.8, *P* < 0.0001; rmANOVA; *P* < 0.01 or 0.001 except for days 3–4, *post-hoc* Tukey-Kramer-test; Figure 6J] and the patrolling task [genotype × day, *F*_(4, 128)_ = 5.38, *P* = 0.0005; rmANOVA; *P* < 0.05 or 0.01 except for days 1–2, *post-hoc* Tukey-Kramer-test; Figure 6L].

Finally, reaction time and delay discounting were tested. During the training phase of the reaction time task, mHb:DTA mice missed slightly more rewards than controls. During testing (with premature responses preventing reward), the mice overcame this deficiency but made more premature and escape responses compared with controls [genotype, *F*_(1, 31)_ = 7.42, *P* = 0.0105; genotype × day, *P* = 0.18, rmANOVA; Figure 6M] [genotype × delay, *F*_(2, 62)_ = 3.54, *P* = 0.035, rmANOVA; *P* < 0.01 delay 1.5 and 2.5 s, *post-hoc* Tukey-Kramer-test; Figure 6N], indicative of impaired inhibitory control. Correct response latency was normal during all phases. During the training phase of the delay discounting task, mHb:DTA mice exhibited a normal saccharin preference and rapidly reached near maximum values (genotype, ns, rmANOVA; Figure 6O). Both groups drank more in response to saccharin exposure, but the increase was larger in mHb:DTA mice [genotype × day, *F*_(3, 93)_ = 12.15, *P* < 0.0001, rmANOVA; *P* < 0.01 at days 1–5, *post-hoc* Tukey-Kramer-test; Figure 6P]. During the testing phase with increased delays to saccharin preference, mHb:DTA mice showed greater resistance to abandoning saccharin, making many compulsive nose-pokes at the closed saccharin door [genotype × day, *F*_(2, 62)_ = 7.17, *P* = 0.0016, rmANOVA; *P* < 0.001 at 0.5–7.5 s and 8 s delays, *post-hoc* Tukey-Kramer-test; Figure 6Q] [genotype × day, *F*_(2, 62)_ = 5.71, *P* = 0.0053, rmANOVA; *P* < 0.01 or 0.05 at 0.5–7.5 s or 8 s delays, *post-hoc* Tukey-Kramer-test; Figure 6R]. These concomitant observations indicated that increased resistance in these mice is very unlikely to result in improved choice control, but is more indicative of a poorly controlled response to saccharin reward, perhaps redolent of the excessive nicotine intake of rats with Hb manipulations (Fowler et al., 2011).

Thus, mHb:DTA mice exhibited maladaptation, hyperactivity, impaired spatial memory, deficits in flexible learning, and high levels of impulsive/compulsive behaviors. These results from female mice in a social setting were consistent with those of male mice in the conventional behavioral tasks.

### Attenuated responses of mHb:DTA mice to systemic administration of nicotine

**Figure 7:**
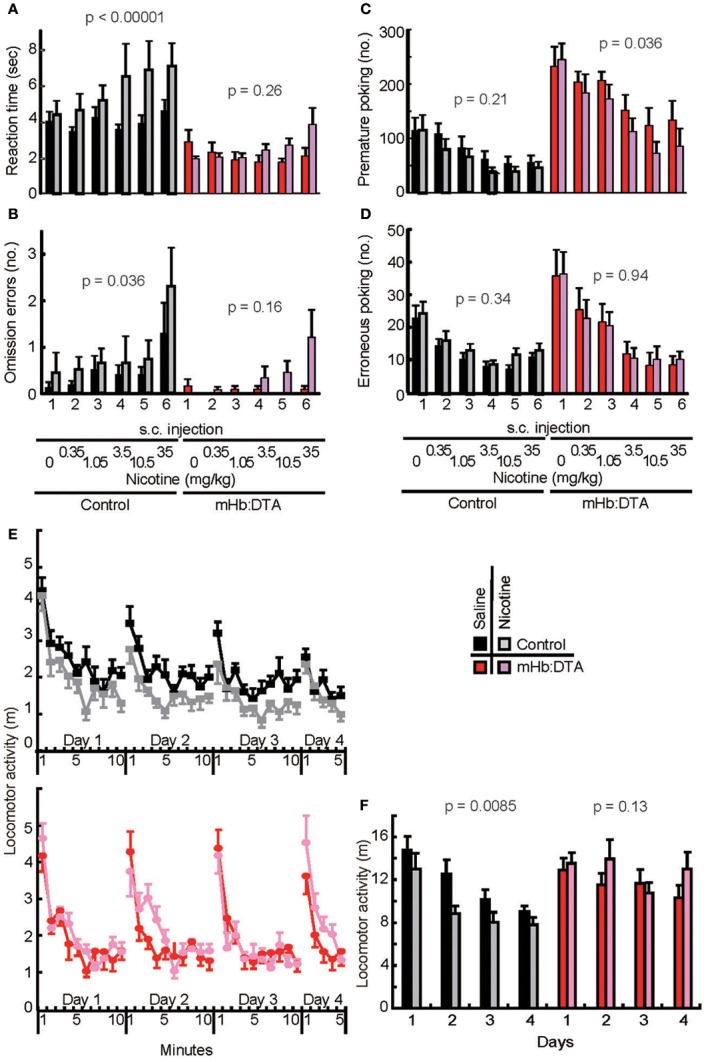
**Lack of susceptibility to nicotine in mHb:DTA mice on 5-CSRTT performance and adaptation to a new environment. (A–D)** Effects of nicotine on 5-CSRTT performance (mice were 9–11 months old, control-saline *n* = 9, control-nicotine *n* = 7, mHb:DTA-saline *n* = 7, mHb:DTA-nicotine *n* = 8). **(A)** Reaction times for nose-pokes after the presentation of signal light in 5-CSRTT. Nicotine (expressed as a free base dose) administration increased reaction times in a dose-dependent manner in control [nicotine main effect within subject *F*_(1, 162)_ = 17.4, Three-Way ANOVA] but not mHb:DTA mice [nicotine main effect within subject *F*_(1, 162)_ = 1.28]. **(B)** Omission errors were modestly increased by nicotine administration in control [nicotine main effect *F*_(1, 162)_ = 4.48, Three-Way ANOVA] but not mHb:DTA mice [nicotine main effect *F*_(1, 162)_ = 2.02]. **(C)** High premature nose-poke rates in mHb:DTA mice were modestly reduced by nicotine administration [nicotine main effect *F*_(1, 162)_ = 1.54 and 4.6 within control and mHb:DTA, respectively, Three-Way ANOVA]. **(D)** Erroneous nose-pokes were not altered by nicotine [nicotine main effect *F*_(1, 162)_ = 0.94 and 0.005 within control and mHb:DTA, respectively, Three-Way ANOVA]. **(E,F)** Effects of nicotine on the new environment adaptation task (mice were 6–7 months old, *n* = 8/group). Figure shows locomotor activity per 1 min **(E)** and for the first 5 min **(F)**. Control mice showed decreased locomotor activity and habituated to the environment after repeated exposures. This habituation was accelerated by nicotine [nicotine main effect within wild-type control *F*_(1, 112)_ = 7.18, Three-Way ANOVA]. mHb:DTA mice did not show typical habituation, and nicotine had no significant effect on locomotor behavior in any session [nicotine main effect within mHb:DTA *F*_(1, 112)_ = 2.21]. s.c., subcutaneous. Data represent mean ± SEM.

Among the abnormalities mentioned above, maladaptation and high impulsivity/compulsivity were the most prominent features. Cells of the mHb are highly sensitive to nicotine (De Biasi and Salas, 2008), and stimulation of cells that uniquely express α 3 β4 nicotinic receptors results in the release of acetylcholine in the IPN (Grady et al., 2009). Moreover, it is well known that patients with various psychiatric disorders, including schizophrenia and depression with comorbid impulsivity, tend to consume tobacco, most likely as a form of self-medication (Dani and Harris, 2005; Kumari and Postma, 2005; Lawrence et al., 2009). We hypothesized that this reflects hypofunction within the mHb-IPN pathway. To test this proposed causal link between behavioral phenotypes and the cholinergic mHb–IPN pathway, we examined the effects of systemically administered nicotine. We used modest doses of nicotine, up to 35 μg/kg (freebase), to mimic the condition of smoking a single cigarette, assuming a body weight of 60 kg, 3 mg nicotine/cigarette, and an intake of 70%. In the 5-CSRTT tests, control mice injected with nicotine at doses of 3.5 μg/kg or more exhibited delayed nose-pokes at the signaled hole [genotype, *F*_(1, 162)_ = 34.37, *P* < 0.0001; nicotine *F*_(1, 162)_ = 13.83, *P* = 0.0003; genotype × nicotine, *F*_(1, 162)_ = 4.39, *P* = 0.038; genotype × nicotine × dose/day, *F*_(5, 162)_ = 0.21, *P* = 0.96; nicotine main effect within control (*F*_(1, 162)_ = 17.37, *P* < 0.00001) and mHb-DTA (*F*_(1, 162)_ = 1.28, *P* = 0.26), Three-Way ANOVA; Figure 7A]. These data indicated that the mHb:DTA mice exhibited no significant delays at any of the tested nicotine doses. The number of omission errors was increased in nicotine-injected control mice, but not in mHb:DTA mice [genotype, *F*_(1, 162)_ = 12.46, *P* = 0.0005; nicotine *F*_(1, 162)_ = 6.23, *P* = 0.014; dose/day *F*_(5, 162)_ = 5.57, *P* < 0.0001; nicotine main effect within control (*F*_(1, 162)_ = 4.48, *P* = 0.036) and mHb-DTA (*F*_(1, 162)_ = 2.02, *P* = 0.16), Three-Way ANOVA; Figure 7B]. Nicotine modestly decreased premature responses [genotype, *F*_(1, 162)_ = 75.02, *P* < 0.00001; nicotine, *F*_(1, 162)_ = 5.781 *P* = 0.017; genotype × nicotine, *F*_(1, 162)_ = 0.45, *P* = 0.5, genotype × nicotine × dose/day, *F*_(5, 162)_ = 0.2, *P* = 0.96; nicotine main effect within control (*F*_(1, 162)_ = 1.54, *P* = 0.21) and mHb:DTA (*F*_(1, 162)_ = 4.61, *P* = 0.036), Three-Way ANOVA; Figure 7C]. No nicotine effect on the number of erroneous nose-pokes was observed for either genotype [genotype, *F*_(1, 162)_ = 11.12 *P* = 0.0011; nicotine *F*_(1, 162)_ = 0.37, *P* = 0.54, genotype × nicotine × dose/day, *F*_(5, 162)_ = 0.04, *P* = 0.999; nicotine main effect within control (*F*_(1, 162)_ = 0.91, *P* = 0.34) and mHb:DTA (*F*_(1, 162)_ = 0.55, *P* = 0.94), Three-Way ANOVA; Figure 7D]. In the new environment-adaptation experiments, nicotine administration (35 μg/kg) accelerated adaptation in control mice, within a single session and across repeated sessions [genotype, *F*_(1, 112)_ = 8.43, *P* = 0.0044; genotype × nicotine, *F*_(1, 112)_ = 8.69, *P* = 0.0039, genotype × nicotine × day, *F*_(3, 112)_ = 0.82, *P* = 0.49; nicotine main effect within controls (*F*_(1, 112)_ = 7.18, *P* = 0.0085) and mHb:DTA (*F*_(1, 112)_ = 2.21, *P* = 0.14), Three-Way ANOVA; Figures 7E,F]. Nicotine administration in mHb:DTA mice failed to induce any effect in this test. Taken together, these data confirm that the mHb–IPN pathway is a central circuit underlying inhibitory control and environmental adaptation, which are major phenotypes of mHb-DTA mice.

### c-Fos mapping

**Figure 8:**
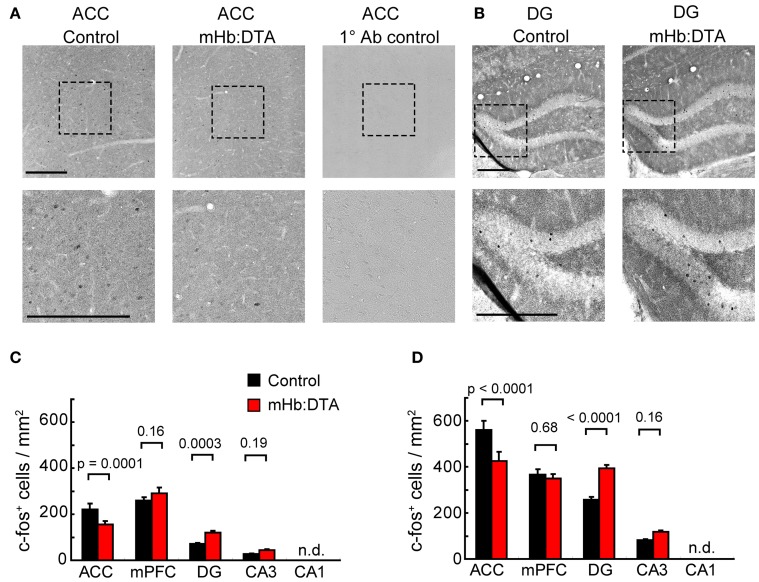
**Immunohistochemical analyses of c-Fos expression.** Both mHb:DTA and control mice were trained in the 5-CSRTT up to spatial session 2. Ninety minutes after the first trial of the last session, the brains were fixed by perfusion and processed for immunohistochemical analyses. **(A,B)** Representative images of the anterior cingulate cortex (ACC, **A**) and dentate gyrus (DG, **B**) are shown. Lower panels represent high magnification image of the square dashed line. Right panels in **(A)** represent negative controls without primary antibodies. **(C)** The number of c-Fos-positive cells in mHb:DTA mice trained was significantly lower in the ACC and higher in the DG and CA3 [*N* = 4 mice, *n* = 16 slices/genotype; Two-Way ANOVA genotype × area interaction, *F*_(3, 280)_ = 10.45, *P* < 0.0001]. n.d., not detected. **(D)** The number of c-Fos-positive cells in mHb:DTA mice exposed to the chamber but not trained was also significantly lower in the ACC and higher in the DG and CA3 [*N* = 4 mice, *n* = 16 slices/genotype; Two-Way ANOVA genotype × area interaction, *F*_(3, 280)_ = 14.9, *P* < 0.0001]. n.d., not detected. *P* value: Bonferroni *post-hoc* test.

To gain insights into the circuit mechanisms underlying high impulsivity, the most prominent phenotype of mHb-DTA mice, we examined c-Fos expression patterns after the 3rd training of spatial session 2 in the 5-CSRTT. Immunohistochemistry for serial sections of whole brains revealed expression of c-Fos in substantial numbers of cells in the medial prefrontal cortex (infralimbic and prelimbic cortex), anterior cingulate cortex (ACC, Figure 8A), and hippocampus (Figure 8B). Interestingly, differential findings were obtained from these brain areas between the genotypes [Two-Way ANOVA, genotype × area interaction. *F*_(3, 280)_ = 10.45; Bonferroni between genotypes *P* = 0.0001, 0.16, 0.0003, and 0.19 for ACC, mPFC, DG and CA3, respectively; Figure 8C]. We observed smaller numbers of c-Fos positive cells in the ACC of mHb-DTA mice, with no differences between genotypes in the medial prefrontal cortex. In contrast, we observed larger numbers of c-Fos positive cells in dentate gyrus (DG) of the mHb-DTA mice. We did not detect c-Fos positive cells in hippocampal area CA1 of either genotype. Another group of mice that was trained in parallel with the mice mentioned above was exposed to the same chamber without training in the last session. These mice showed larger numbers of c-Fos positive cells, probably reflecting the novel condition [Two-Way ANOVA, genotype × area interaction, *F*_(3, 280)_ = 14.9; Bonferroni between genotypes *P* < 0.0001, = 0.68, < 0.0001, and = 0.16 for ACC, mPFC, DG and CA3, respectively; Figure 8D]. Interestingly, the ratios between genotypes were maintained under both conditions. Thus, these differential results reflect differences in the genotype, and suggest a crucial involvement of the ACC and hippocampus in the behavioral abnormalities of mHb-DTA mice.

## Discussion

The physiologic functions of the mHb in mammals are unclear. To examine this issue, we generated mice in which the mHb–IPN pathway was selectively ablated in late postnatal stages, thereby minimizing developmental abnormalities. The results of our extensive behavioral studies indicated that the mHb–IPN pathway plays a crucial role in various behavioral domains, particularly inhibitory control and cognition-dependent executive functions. These data support previous findings suggesting the involvement of the mHB–IPN pathway in various mental disorders, such as schizophrenia and attention-deficit/hyperactivity disorder.

Within traditional experimental paradigms, mHb:DTA mice exhibited abnormalities in various behavioral domains, such as anxiety, locomotor activity, habituation, sensorimotor gating, spatial memory, impulsive/compulsive behaviors, and decision-making. The behavioral phenotypes of the mHb:DTA mice closely resembled phenotypes of rodents with classic bilateral lesions of the entire habenular complex, or cuts to the fasciculus retroflexus (Murphy et al., 1996; Lecourtier et al., 2004, 2005; Lecourtier and Kelly, 2005; Heldt and Ressler, 2006). The methods used in the previous studies failed to discriminate between the mHb and lHb or between the habenula and passing axons, preventing specific analysis of mHb function. In the present study, the mHb:DTA mice carry a highly selective ablation in the mHb–IPN pathway. Although there is some cell death in the M2 subset of the lHb and small fractions of the PVT and ReT, it is unlikely that the small cell loss in these areas is responsible for the profound loss of nicotine responsiveness observed in the 5-CSRTT and adaptation tests, which are key findings that help to explain the phenotypic observations. In addition, to the best of our knowledge, there is no evidence of nicotinic acetylcholine receptor expression in the M2 subset of the lHb. Thus, the lack of susceptibility of mHb:DTA mice to systemic nicotine administration in these tests strongly supports a role for the mHb–IPN pathway, which expresses a high level of unique nicotinic acetylcholine receptors. These data therefore suggest a higher profile for the mHb–IPN pathway than previously thought.

IntelliCage studies using female mice revealed behavior congruent with the observations from the conventionally tested males, confirming the transgenic phenotypes. The mHb:DTA mice showed higher premature responses in a delay-dependent manner and perseverative nose-pokes toward closed doors for saccharin, indicating increased impulsivity and compulsivity. The mice exhibited maladaptation to new environments and eventual hyperactivity throughout the session. Moreover, the study clearly illustrated deficits in reversal learning and highly demanding cognitive learning paradigms requiring short-term memory. These data strengthen our understanding of the behavioral characteristics of mHb:DTA mice in combination with the data from conventional testing paradigms and emphasize the utility of testing mice living in social groups with minimal human interference (Krackow et al., 2010; Voikar et al., 2010).

Pathologic impulsivity and compulsivity are associated with various psychiatric and personality disorders (Pattij and Vanderschuren, 2008; Torregrossa et al., 2008), including schizophrenia, attention deficit/hyperactivity disorder, obsessive–compulsive disorder, and drug abuse. While impulsivity and compulsivity represent distinct neurocognitive functions, they are often comorbid, suggesting causal links between these behaviors and overlaps in the responsible circuits (Belin et al., 2008; Torregrossa et al., 2008; Fineberg et al., 2010). Here, mHb:DTA mice exhibited high rates of premature nose-pokes in the 5-CSRTT from the early stages of training, maladaptation to new environments, and aversions of both delay and effort in decision-making tests. Compulsivity is highly associated with deficits in reversal learning and hyperactivity, particularly under rewarded conditions. Our results from mice with deficits in a selective circuit explain why abnormalities in the behavioral domains mentioned above tend to be comorbid.

Recent studies with zebrafish suggest a role for the septum–mHb–IPN pathway in fear responses (Agetsuma et al., 2010; Jesuthasan, 2012). The results of our open-field mouse study and elevated plus-maze tests partly support a modest role for this pathway in anxiety-related behaviors. We observed no differences in fear conditioning and avoidance learning to the air puffed corner, however, in the IntelliCage system. Based on these findings, we suggest that the mHb–IPN pathway in mice plays a central role in inhibitory control to prevent impulsive and compulsive behaviors. It is highly likely that deficits in these domains underlie other cognitive phenotypes.

The lHb sends efferents directly to serotonergic and dopaminergic centers (Klemm, 2004; Lecourtier and Kelly, 2007). Recent studies have revealed additional pathways to dopaminergic centers from the lHb, through the rostromedial tegmental nucleus (Jhou et al., 2009). Efferents from the mHb solely innervate the IPN, and the IPN sends efferents to the dopaminergic, serotonergic, and noradrenergic centers through the laterodorsal tegmental area (Groenewegen et al., 1986). Thus, deficits in cholinergic mHb inputs affect downstream monoaminergic centers and related structures. The findings of this study indicate a crucial role for the circuitry of mHb–IPN–monoaminergic centers in several behavioral domains, such as impulse control and adaptation. The integrated functioning of mHb–IPN–monoaminergic centers and the lHb-mediated pathway might be essential for controlling monoaminergic centers in a state-dependent manner. The precise mechanisms underlying the coordination of these dual pathways remain to be elucidated. As a part of the downstream mechanism, the results of c-Fos immunostaining from mHb:DTA mice trained in the early stages of 5-CSRTT suggest hypofunction of the ACC and hyper-responsiveness in DG regions of the hippocampus in mHb-DTA mice (Figure 8). c-Fos positive cells were more abundant in mice exposed to the chamber without training on the last day with no differences in the ratio between genotypes. Thus, the increases may reflect subtle changes in the context. We hypothesize that hypofunction of the ACC and the hyper-responsiveness of the hippocampal DG underlie the maladaptive behavior. This may in part explain the impulsive behaviors. ACC lesions increase anticipation and perseverative responses in the 5-CSRTT (Muir et al., 1996), and the hippocampal CA3 and DG regions are strongly associated with novelty detection (Frank et al., 2004; Procaccini et al., 2011).

Further studies focusing on the upstream and downstream mechanisms of the mHb–IPN pathway (Procaccini et al., 2011) under different experimental conditions will help to elucidate how inhibitory control is achieved and may provide effective therapeutic strategies for various mental disorders. The mHb-DTA mice will be valuable for investigating therapeutic strategies for a subset of psychiatric disorders with underlying habenular hypofunction.

## Author contributions

Yuki Kobayashi and Shigeyoshi Itohara designed the research; David P. Wolfer designed the IntelliCage experiment; Yuki Kobayashi, Yoshitake Sano, Elisabetta Vannoni, Hiromichi Goto, Hitomi Suzuki, Atsuko Oba, and Toshio Ikeda conducted the research; Yuki Kobayashi, Yoshitake Sano, Hiromichi Goto, Niall P. Murphy, David P. Wolfer, and Shigeyoshi Itohara analyzed the data; and Yuki Kobayashi, Yoshitake Sano, Hiromichi Goto, Niall P. Murphy, Hiroaki Kawasaki, Shigenobu Kanba, Hans-Peter Lipp, David P. Wolfer, and Shigeyoshi Itohara wrote the paper.

### Conflict of interest statement

The authors declare that the research was conducted in the absence of any commercial or financial relationships that could be construed as a potential conflict of interest.
